# Combined extracts of *Moringa oleifera*, *Murraya koeingii* leaves, and *Curcuma longa* rhizome increases energy expenditure and controls obesity in high-fat diet-fed rats

**DOI:** 10.1186/s12944-020-01376-7

**Published:** 2020-08-28

**Authors:** Sreenath Kundimi, Krishna Chaitanya Kavungala, Swaraj Sinha, Venkata Narasimha Rao Tayi, Nagendra Rao Kundurthi, Trimurtulu Golakoti, Barbara Davis, Krishanu Sengupta

**Affiliations:** 1 Laila Nutraceuticals R&D Center, JRD Tata Industrial Estate, Kanuru, Vijayawada, Andhra Pradesh 520007 India; 2 PLT Health Solutions, Inc., Morristown, NJ USA

**Keywords:** Adipokine, Diet-induced obesity, LI85008F, Resting energy expenditure, Uncoupling protein-1

## Abstract

**Background:**

LI85008F is a proprietary combination of leaf extracts of *Moringa oleifera*, *Murraya koeingii*, and extract of *Curcuma longa* rhizome. This herbal extract combination is an effective weight loss supplement for overweight and obese subjects. The present study aimed to investigate the thermogenic potential of the LI85008F in high-fat diet (HFD)-induced obese *Sprague Dawley* rats.

**Methods:**

Seven rats received a regular diet (RD), and twenty-one rats received a high-fat diet (HFD) for 56 days. On day 28, the HFD-fed rats were randomized into three groups (*n* = 7). Starting from day 29 through day 56, one HFD-fed group received daily oral gavage of 0.5% Carboxymethylcellulose Sodium (CMC) alone (HFD), and the remaining two groups received 100 and 250 mg/kg LI85008F (LI85008F-100 and LI85008F-250, respectively).

Body weight, fat mass, fat cell size, liver weight, liver triglyceride were measured. The energy metabolism parameters were measured using indirect calorimetry. In serum, the metabolic and endocrine markers were analyzed. The adipogenic and thermoregulatory proteins expression in the white adipose tissue (WAT) were analyzed using an immunoblot assay.

**Results:**

Supplementation with both doses of LI85008F significantly increased resting energy expenditure (REE) in the obese rats. The LI85008F-250 rats showed significant up-regulation of uncoupling protein-1 (UCP-1) expression, as compared with the HFD rats. LI85008F significantly reduced body weight gain, fat mass, fat cell size, liver weight, and hepatic triglycerides. Serum triglyceride, total cholesterol, glucose, leptin, and fat cell markers were significantly reduced in LI85008F-supplemented rats compared to the HFD rats.

**Conclusion:**

The present data suggest that LI85008F reduces body fat mass and controls body weight gain via increasing energy metabolism in combination with reduced lipogenesis in diet-fed obese rats.

## Background

Obesity is a state of metabolic disorder resulting in an abnormal or excess storage of fat in the body, and it is a potential risk factor for diabetes, cardiovascular diseases, cancer, and other chronic diseases [1]. The World Health Organization (WHO) criteria classify an overweight individual with a Body Mass Index (BMI, kg/m^2^) between 25 and 29.9 and obese with a BMI of 30 and above [1]. Globally, the growing prevalence of obesity estimates that almost half of the adult population will be overweight or obese by 2030 [2].

In the majority of cases, dietary habits and sedentary lifestyle are predisposing factors for excess fat accumulation in the body [3]. Chronic caloric intake that exceeds daily energy expenditure results in an increase in the size and number of fat cells resulting in the onset or progression of obesity [4]. Identifying strategies to increase energy expenditure could prove important for the management of body fat. A series of clinical studies have established that routine physical exercise, consumption of thermogenic food or dietary supplements provide significant benefit in reducing body fat and improve the body composition in overweight or obese subjects [5–8].

LI85008F is an herbal extract combination composed of aqueous-ethanol extract of *Moringa oleifera* leaves, *Murrya koenigii* leaves, and *Curcuma longa* rhizomes extract standardized to not less than 95% total curcuminoids [9, 10]. This composition showed synergy in inhibiting new adipocyte formation through the PPARγ dependent cell differentiation process in vitro and demonstrated that LI85008F significantly increased lipid breakdown in cultured fat cells [11]. Subsequently, two clinical studies have demonstrated that LI85008F supplementation significantly reduced body weight (BW), along with BMI in overweight and obese human subjects. LI85008F also improved the lipid profile and adiponectin level in serum, compared with the placebo [9–11]. Two exciting findings in the earlier clinical studies were enhanced intracellular fat breakdown in the LI85008F treated 3 T3-L1 fat cells, and increased serum adiponectin level in LI85008F supplemented participants. These observations prompted to postulate whether LI85008F might trigger thermogenic activity in the body [9–11]. Earlier animal studies have demonstrated that adiponectin increases energy expenditure via activation of thermogenesis in brown fat cells and suggest this hormone is an essential regulator of energy metabolism [12, 13].

The primary objective of the present study was to evaluate whether LI85008F increased the energy expenditure at resting conditions (REE) in conjunction with reduced adiposity in the obese rats. Further, the effects of LI85008F supplementation on fat cell markers in the adipose tissue, essential modulators of energy metabolism in serum, and the critical thermoregulatory Uncoupling protein 1 (UCP-1) expression were evaluated in the WAT of the obese rats.

## Materials and Methods

### Study material

LI85008F is a composition of six parts *Moringa oleifera* leaf aqueous-ethanol extract, three parts *Murraya koenigii* leaf aqueous-ethanol extract, and one part *Curcuma longa* rhizome extract standardized to not less than 95% total curcuminoids. Fresh plant raw materials collected from local areas in Andhra Pradesh, India. The Taxonomy Division of Laila Impex R&D Centre, Vijayawada, India, preserved their voucher specimens. Earlier communications described the methods of individual extract preparation from the dried plant raw materials and phytochemical standardization of LI85008F formulation [9–11]. The final standardized product contains at least 7.0% total curcuminoids (Curcumin + Bisdemethoxycurcumin + Demethoxycurcumin), 0.1% Mahanine, and 0.2% Quercetin 3-O-glycoside. The reference standards Quercetin 3-Glucoside (cat.# 16,654), Curcumin (cat.# 08511), Bisdemethoxycurcumin (cat.# 90,594), Demethoxycurcumin (cat.# D7696) (Sigma-Aldrich, St. Louis, Missouri, United States), and Mahanine (LI10704, 99%, Laila Nutraceuticals in-house standard) were used to identify the peaks in High-Performance Liquid Chromatography (HPLC), for standardization of LI85008F. Laila Nutraceuticals, a CGMP-certified manufacturing facility at Vijayawada, India, produced LI85008F. LI85008F is commercially available as Slimvance® or Slendacor®.

### Animal care and experimental design

The present study utilized male *Sprague Dawley* rats of 6–8 weeks age (150–200 g). The rats were purchased from Palamur Biosciences Private Limited, Hyderabad, Telangana, India. They were acclimatized to the standard laboratory conditions (23 ± 2 °C temperature and 40–70% relative humidity and 12 h light/dark cycle) for 7 days. During acclimatization, the animals received a standard rodent pellet diet (VRK Nutritional Solutions Pvt. Ltd., Pune, India) and mineral water ad libitum. Each one-hundred g of the standard pellet diet or the regular diet (RD) contained 60 g carbohydrates, 18 g proteins, 4 g fat, 4 g fiber, 1.5 g mineral mix. The Institutional Animal Ethics Committee (IAEC) of Laila Impex, Vijayawada, India approved the experimental protocol (Protocol No: LI/IAEC/LI190104). The animal care and experimental procedures followed the guidelines of the Committee for the Purpose of Control and Supervision of Experiments on Animals (CPCSEA), India.

This study consisted of two phases, i.e., an induction phase for obesity development followed by a supplementation or treatment phase. Each phase was 28 days long. In the induction phase, seven animals received a regular diet (RD) (G1; *n* = 7), and thirty-five animals received a high-fat containing diet (HFD) (cat.# 104,641; 5.34 kcal/g, Dyets Inc., Bethlehem, Pennsylvania, United States). Each one-hundred g of HFD contained 20 g casein, 18.6 g sucrose, 15 g maltose dextrin, 36 g anhydrous milk fat, 3.2 g cellulose, 3.5 g mineral mix# 210025, 1 g vitamin mix# 310025, 1.18 g sodium bicarbonate, 0.77 g potassium citrate. At the end of the induction period, twenty-one animals were selected based on the maximum effect on their BW obtained from the HFD feed. The selected obese rats were randomly allocated into three groups (*n* = 7); G2 (HFD), G3 (HFD + 100 mg/kg LI85008F), and G4 (HFD + 250 mg/kg LI85008F). From day 29, the start of the supplementation phase, the rats received an oral gavage of 0.5% Carboxymethylcellulose Sodium (CMC) alone (G1 and G2) or combined with the selected doses of LI85008F (G3 and G4). The low dose of the herbal blend tested in the present study was approximately equivalent to the clinical dose of LI85008F [10, 11].

### Body and organ weight measurements

The BW of the overnight fasted animals were recorded every week during the study, using an electronic weighing balance (Model# RW00–1220-044; Mettler-Toledo, Columbus, Ohio, United States). The difference between the amount of leftover food and the supplied food estimated their daily food intake.

On day 56, following CO_2_ euthanasia, the liver and adipose tissue (from the abdominal area, inguinal, epididymis, mesenteric, and inter-scapular) were collected from the experimental rats. The excised tissues were weighed using an analytical balance (Model# CP224S; Sartorius, Göttingen, Germany). The tissues were stored at − 80 °C in aliquots until analysis.

### Indirect calorimetry

A four-chamber indirect calorimetric system containing Oxylet Pro 4 Animal Rat/Mice Metabolic Monitoring System (Model# LE1332) equipped with a gas analyzer (cat# LE405) procured from Harvard Apparatus (Cornella, Spain) measured the energy metabolism parameters of the experimental rats. At the end of the study, overnight fasted rats were placed in individual metabolic chambers maintained at 24 °C. The rats were acclimatized in the chamber for 2 h before the start of any measurement. The oxygen (O_2_) and carbon dioxide (CO_2_) analyzers were calibrated with high-purity gases, supplied by the vendor. A computer-assisted data acquisition program METABOLISM V2.2.01 (Harvard Apparatus, Cornella, Barcelona, Spain) recorded the volumes of O_2_ and CO_2_ exchanges over 4 h at 1 min interval. The average volumes of CO_2_ exhaled, and O_2_ utilized per min were normalized with their respective BW, and presented as VCO_2_ and VO_2_ in mL/min/kg BW, respectively. The different metabolic parameters, viz. respiratory exchange ratio (RER), rate of fat and carbohydrate oxidation (g/min/kg), and resting energy expenditure (REE; joule/min/kg BW) were calculated following the modified Weir equation [14], considering one Kcal equal to 4184 joules (J).

$$ \mathrm{REE}\left(\mathrm{Kcal}/\min /\mathrm{kg}\right)=\left[3.941\left({\mathrm{VO}}_2\right)+1.106\left({\mathrm{VCO}}_2\right)\right] $$

### Histopathology

The epididymal fat tissue was fixed in 10% neutral buffered formalin for 48 h and paraffin-embedded following the standard method [15]. The paraffin-embedded tissue blocks were sectioned at a thickness of 4 μm using a manual rotary microtome (Microtome RTS2125, Leica Biosystems, Mumbai, India). The tissue sections were deparaffinized and processed in graded alcohol, stained with hematoxylin and eosin (H&E) following the standard procedure [16]. The stained tissue sections were examined at 200X magnification under an Axio Scope A1 microscope (Carl Zeiss GmbH, Jena, Germany). A CCD camera (ProgRes C5, Genoptik, Jena, Germany) captured the bright-field images. The cross-sectional area of at least five-hundred randomly selected fat cells in each tissue section was calculated using automated software, Adiposoft (Image J, NIH, Bethesda, Maryland, United States).

### Biochemical indices

At the end of the study, the overnight fasted animals were anesthetized with isoflurane. Blood samples were collected from retro-orbital plexus, and the sera were separated by centrifuging the blood samples at 3200×*g* for 15 min at 4 ± 2 °C, stored at − 80 °C in aliquots. Alanine aminotransferase (ALT; cat.# 0018257440), aspartate aminotransferase (AST; cat.# 0018257540), total cholesterol (TC; cat.# 0018250540), and triglyceride (TG; cat.# 0018258740) assay kits were procured from Instrumentation Laboratory, New Delhi, India. Glucose assay kit (GL; cat.# MLGL500) was purchased from Microlyn Healthcare Private Limited, Chennai, Tamil Nadu, India. The test procedures were following the protocol provided in the kit application manuals. The assays were performed in an automatic biochemistry analyzer (Model ILab Aries®, Instrumentation Laboratory, Barcelona, Spain).

Commercially available ELISA kits were used to measure the hormones adiponectin, leptin, triiodothyronine (T3), and thyroxin (T4) in serum. The adiponectin (cat.# EZRADP-62 K) and leptin (cat.# EZRL-83 K) ELISA kits were procured from Merck-Millipore (Burlington, Massachusetts, United States); T3 (cat.# T3379T) and T4 (cat.# T4224T-96) assay kits were procured from Calbiotech (El Cajon, California, United States). The assays were performed according to the manufacturer’s instructions. Briefly, the serum samples were incubated in duplicate wells of capture antibody-coated 96-well microplates. Then, the bound antigen molecules (analytes) were probed with a biotinylated secondary antibody. Following washing of excess free enzyme conjugates, the immobilized antibody-enzyme conjugates were reacted with horseradish peroxidase (HRP) in the presence of the substrate 3,3′,5,5′-tetramethylbenzidine. The enzyme activity was measured at 450 nm using a microplate reader (SpectraMax M2, Molecular Devices, San Jose, California, United States). The amount of captured analyte in the serum sample was derived by interpolation from a reference curve generated with the known concentrations reference standards supplied with the kits. Adiponectin and Leptin ELISA kits provided the respective internal standards for quality control checks. The sensitivities of the adiponectin, leptin, T3, and T4 kits were 0.155 ng/mL, 0.04 ng/mL, 0.4 pg/mL, and 2 μg/dL respectively.

A triglyceride assay kit (cat.# 10,010,303; Cayman Chemical, Ann Arbor, Michigan, United States) measured the triglycerides (TG) in the liver tissue lysates. This assay kit uses the enzymatic hydrolysis of TG by lipase to produce glycerol and fatty acids. The glycerol released is subsequently measured by a coupled enzymatic reaction. The sensitivity of the TG assay kit was 1.6 mg/dL. The assay procedure was following the protocol from the manufacturer. Briefly, equal weights of liver tissue samples were minced and homogenized in 2 mL of NP40 substitute assay reagent containing protease inhibitors (cat.# P8340-1ML; Sigma-Aldrich, St. Louis, Missouri, United States) at 4 °C. The tissue homogenates were clarified at 10000×*g* for 20 min at 4 °C. A microplate reader (SpectraMax M2, Molecular Devices, San Jose, California, United States) measured the color at 550 nm. The amount of hepatic TG presented as mg per g of liver tissue.

### Western blotting

The inguinal fat tissues were lysed in CelLytic™ MT cell lysis reagent (cat.# C3228; Sigma-Aldrich, St. Louis, Missouri, United States) in the presence of protease and phosphatase inhibitor cocktails (cat.# P5726; Sigma Aldrich, St. Louis, Missouri, United States). The protein content in the tissue lysates was quantified using the BCA protein assay kit (cat.# 23,225; Thermo Scientific, Waltham, Massachusetts, United States). Twenty micrograms of tissue lysate proteins were loaded on each well and resolved on 10% sodium dodecyl sulfate-polyacrylamide gel at 100 V. The resolved proteins were transferred onto a nitrocellulose membrane at 100 V for 2 h at 4 °C [9]. The blotted membranes were incubated with 1:1000 dilutions of the primary antibodies for 16 h at 4 °C. The antibodies were specific to Activating Protein 2 alpha (aP-2α; cat.# 3208S), CCAAT enhancer-binding protein alpha (C/EBPα; cat.# 2295S) (Cell Signaling Technology, Danvers, Massachusetts, United States), Peroxisome proliferator-activated receptor gamma (PPAR-γ; cat.# SC-271392), cluster of differentiation 36 (CD36; cat.# SC-7309) (Santa Cruz Biotechnology, Dallas, Texas, United States), Perilipin-1 (cat.# PA1–1051), UCP-1 (cat.# PA1–24894) (Thermo Fisher Scientific, Waltham, Massachusetts, United States). After the removal of the excess antibodies, the washed membranes were incubated with 1:10000 dilutions of either HRP-conjugated goat anti-rabbit IgG (H + L) (cat.# 111–035-003) or goat anti-mouse IgG (H + L) (cat.# 115–035-003) for 45 min at room temperature. The enzyme-conjugated secondary antibodies were procured from Jackson ImmunoResearch Laboratories Inc., West Grove, Pennsylvania, United States. Actin protein expression was detected by the β-Actin antibody (cat.# A2228; Sigma Aldrich, St. Luis, Missouri, United States). A chemiluminescent substrate (cat.# 34,580; Super Signal West Pico PLUS, Thermo Scientific, Waltham, Massachusetts, United States) developed the protein bands and a Bio-Rad Chemi Doc XRS+ imager (Bio-Rad, Hercules, California, United States) captured the protein band images. Densitometry analysis of the protein bands was performed using Carestream molecular imaging software v.5.0.2.30 (Carestream Health, Rochester, New York, United States).

### Statistical analysis

The data present as mean ± SD. Data were analyzed using GraphPad Prism software v5.01 (GraphPad Software, Inc., San Diego, California, United States). One-way ANOVA, followed by Dunnett’s test, analyzed the intergroup comparisons; *P* < 0.05 was considered significant.

## Results

### LI85008F mitigates body weight gain in high-fat diet-fed obese rats

**Fig. 1:**
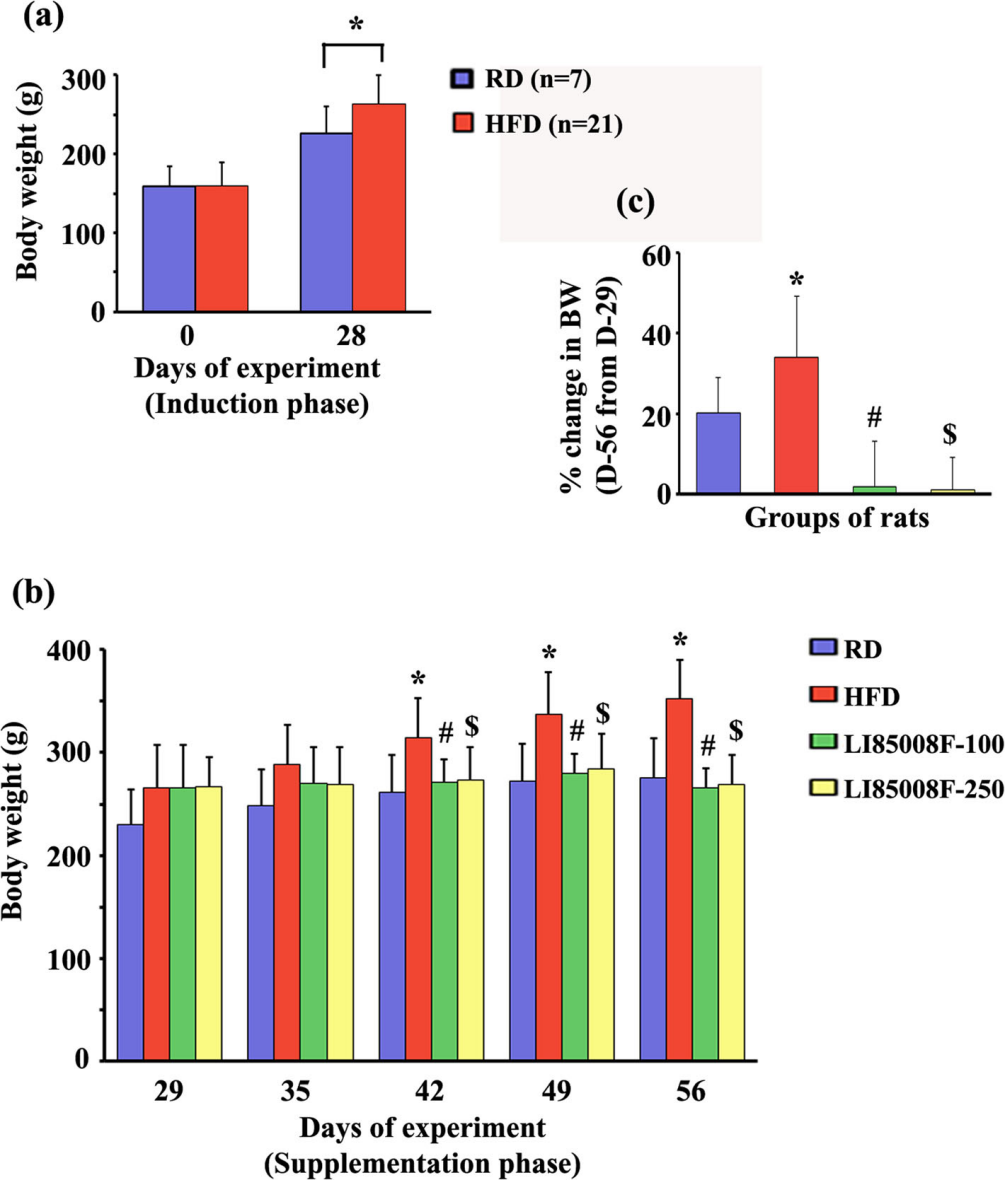
LI85008F regulates body weight and reduces body weight gain in high-fat diet-fed obese rats. **a** The bar graph shows changes in body weights (mean ± SD) of *Sprague Dawley* rats receiving regular diet (RD; *n* = 7) or a high-fat diet (HFD; *n* = 21) for 28 days of the induction phase of the study. **b** The bar graph presents the effect of LI85008F supplementation on the body weights of the experimental rats during the 28-day supplementation period (from day 29 through day 56). Body weights measured weekly, and the data present the mean ± SD of body weights (*n* = 7). The rats received either a regular diet (RD) or a high-fat diet (HFD) or HFD in combination with a daily dose of 100 mg/kg (LI85008F-100) or 250 mg/kg (LI85008F-250) LI85008F, as described in the Materials and Methods. **c** The bar diagram presents the mean ± SD of percent changes (from day 29) in body weight (BW) at day 56. *, # and $ indicate significance (*P* < 0.05) in RD vs. HFD, HFD vs. LI85008F-100, and HFD vs. LI85008F-250 comparison analysis, respectively using one-way ANOVA

At the end of the 28-day induction phase, the HFD rats showed a significant gain in BW compared with the RD rats (Fig. 1a). The difference between the BW gain in the selected obese rats (*n* = 21) and the RD rats (*n* = 7) was statistically significant (68.86 ± 18.22% vs. 45.44 ± 4.55; *P* < 0.05). Twenty-eight days of LI85008F supplementation showed gradual reductions of BW gain in the HFD rats (Fig. 1b). The effect (*P* < 0.05) of LI85008F started from day 42, i.e., 14 days of supplementation, when compared with the HFD obese rats (Fig. 1b). On day 56, LI85008F-100 and LI85008F-250 rats showed mean BW gains of 1.68% (*P* < 0.05) and 1.05% (*P* < 0.05), respectively, in comparison, the HFD rats gained 34.02% BW from day 29 (Fig. 1c).

### LI85008F alters energy metabolism in high-fat diet-fed obese rats

Table 1 presents the effect of LI85008F supplementation on various energy metabolism parameters of obese rats. LI85008F-100 and LI85008F-250 rats showed significantly higher VCO_2_, VO_2_, and energy expenditure than the HFD rats. The low and high doses of herbal supplementation yielded 20.22% (*P* < 0.05) and 26.15% (*P* < 0.05) increases in the resting energy expenditure (REE), respectively, compared with the obese rats. Interestingly, LI85008F-100 (14.70% increase vs. G1, *P* < 0.05) and LI85008F-250 (20.36% increase vs. G1, *P* < 0.05) rats also showed significant changes in REE relative to the RD rats as well. The RER was unaltered across the experimental groups. The low and high dose of LI85008F supplemented rats showed enhanced fat oxidation rates by 29.93% (*P* = 0.3732) and 83.67% (*P* < 0.05), respectively, from the HFD rats (Table 1).

**Table 1:** LI85008F alters energy metabolism in high-fat diet-fed obese rats

Metabolic parameters	Groups			
	RD	HFD	LI85008F-100	LI85008F-250
VCO_2_ (mL/min/kg)	15.84 ± 1.70	15.37 ± 2.14	18.40 ± 1.12^#,§^	18.98 ± 1.14^$,§^
VO_2_ (mL/min/kg)	17.09 ± 1.66	16.24 ± 2.04	19.54 ± 1.44^#,§^	20.58 ± 1.08^$,§^
RER (VCO_2_/VO_2_)	0.93 ± 0.03	0.95 ± 0.03	0.94 ± 0.04	0.92 ± 0.03
Fat oxidation (g/min/kg)	2.10 ± 0.87	1.47 ± 0.93	1.91 ± 0.85	2.70 ± 1.03^$^
Carb. oxidation (g/min/kg)	15.55 ± 2.82	16.07 ± 3.45	18.98 ± 1.39^§^	18.32 ± 2.60
Energy expenditure (J/min/kg)	355.1 ± 35.0	338.8 ± 43.3	407.3 ± 28.8^#,§^	427.4 ± 22.6^$,§^

*J* Joule, *RER* respiratory exchange ratio

Data presents mean ± SD; *n* = 7. #, $, and § indicate significance (*P* < 0.05) in HFD vs. LI85008F-100, HFD vs. LI85008F-250, and RD vs. LI85008F-100 or LI85008F-250 intergroup comparison analysis, respectively, using one-way ANOVA

### LI85008F reduces body fat mass and fat cell size in the HFD-fed obese rats

After 28 days supplementation of 100 and 250 mg/kg LI85008F, the rats showed significant reductions of inguinal fat, and epididymal fat masses, compared with the HFD rats (Table 2). Additionally, LI85008F-250 rats showed significant reductions of abdominal and mesenteric fat masses. In comparison with the HFD rats, LI85008F supplemented rats did not show substantial changes in the inter-scapular fat masses (Table 2). Overall, in comparison with the HFD rats, LI85008F-100 and LI85008F-250 rats showed 27.62% (*P* < 0.05) and 30.37% (*P* < 0.05) reductions of total body fat mass, respectively. The total body fat in HFD obese rats increased by 115.62% (*P* < 0.05, vs. G1), compared with the RD rats (Table 2).

**Table 2:** LI85008F reduces adiposity in high-fat diet-fed obese rats

	Groups			
	RD	HFD	LI85008F-100	LI85008F-250
Abdominal fat (g)	2.93 ± 1.13	7.53 ± 0.50^*^	5.60 ± 1.90	5.31 ± 1.30^$^
Inguinal fat (g)	1.79 ± 0.45	4.02 ± 0.75^*^	2.52 ± 0.72^#^	2.43 ± 0.23^$^
Epididymal fat (g)	2.62 ± 0.52	5.57 ± 1.05^*^	3.62 ± 0.46^#^	3.34 ± 0.58^$^
Mesenteric fat (g)	1.14 ± 0.47	2.04 ± 0.44^*^	1.56 ± 0.22	1.44 ± 0.33^$^
Inter-scapular fat (g)	0.39 ± 0.11	0.58 ± 0.18^*^	0.45 ± 0.08	0.43 ± 0.08
Total fat (g)	8.58 ± 2.91	18.50 ± 3.78^*^	13.39 ± 2.93^#^	12.88 ± 2.03^$^
Fat cell size (μm^2^)	1847 ± 379	3107 ± 313^*^	2739 ± 346	2503 ± 412^$^

Data presents mean ± SD; n = 7. *, # and $ indicate significance (*P* < 0.05) in RD vs. HFD, HFD vs. LI85008F-100, and HFD vs. LI85008F-250 intergroup comparison analysis, respectively, using one-way ANOVA

**Fig. 2:**
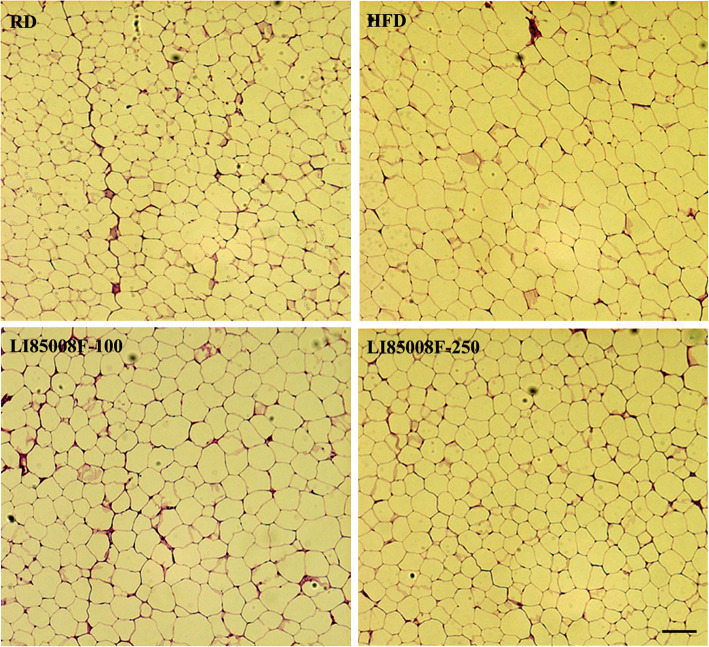
Morphometric analysis of the epididymal fat cells. Representative photomicrographs show Hematoxyline-Eosin stained cross-sections of the epididymal fat tissue of the different groups of animals. The groups of animals are regular diet (RD), high-fat diet (HFD), LI85008F-100 and LI85008F-250, respectively, as described in the Materials and Methods. The bar indicates 100 μm

In parallel, the herbal blend also reduced mean fat cell size, compared with that of HFD rats. Figure 2 depicts representative images of the HE-stained fat tissues from the experimental rats. The morphometric analysis of the fat cells revealed that the fat cell size in LI85008F-100 and LI85008F-250 rats were reduced by 11.85% (*P* = 0.0651) and 19.44% (*P* < 0.05), respectively when compared with the HFD rats (Table 2).

### LI85008F down-regulates expression of key adipogenic marker proteins and up-regulates UCP-1 expression in fat tissue

**Fig. 3:**
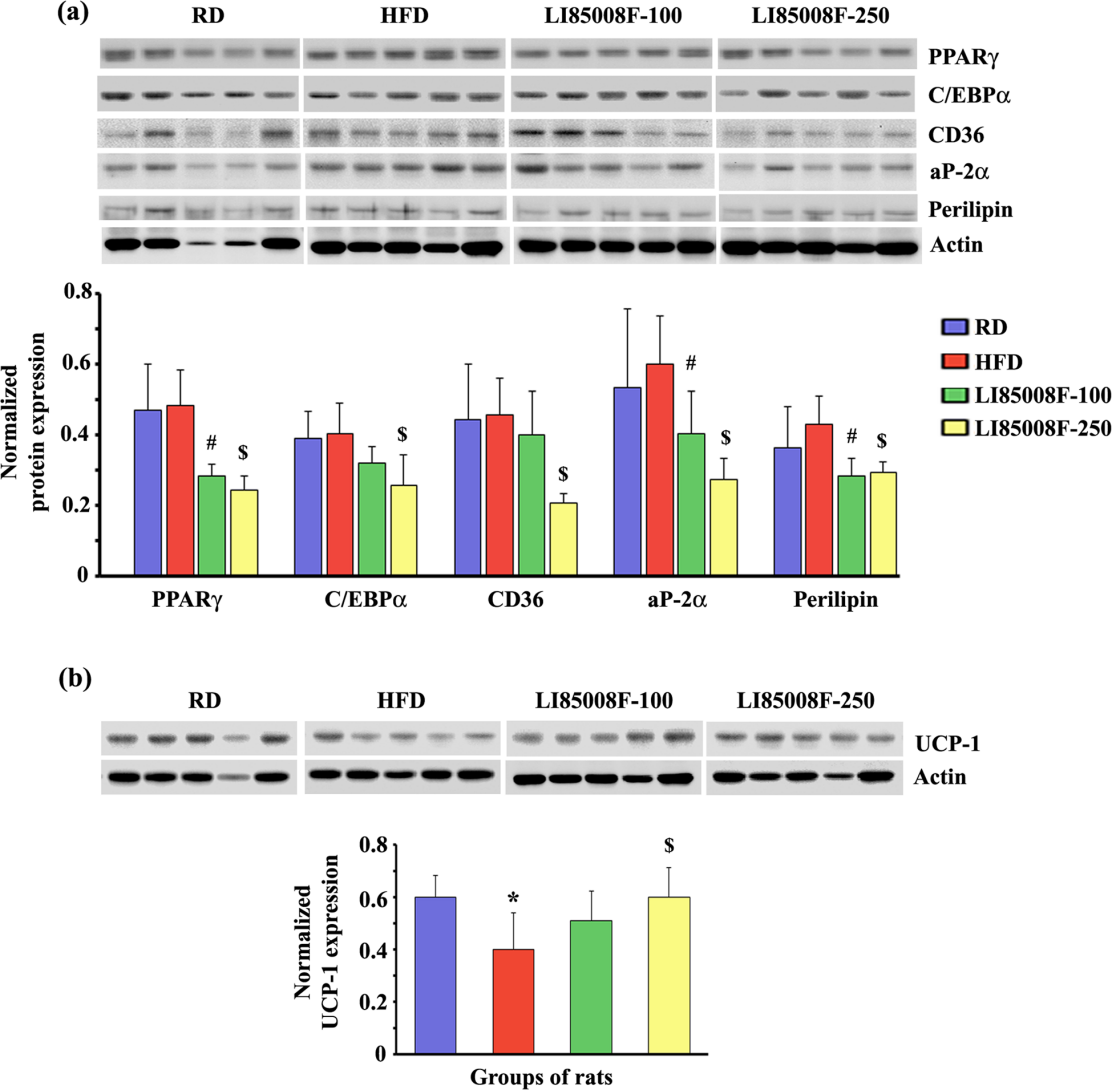
LI85008F modulates the expression of key adipogenic and thermoregulatory proteins in white fat tissue of high-fat diet-fed obese rats. Immunoblot images present the protein expression of key modulators of lipogenesis (**a**) and UCP-1 (**b**) in the inguinal fat tissue of individual animals in different groups, as indicated. In the bar diagrams, each bar presents the mean ± SD of the protein expressions (*n* = 5) normalized with the respective actin expression. The groups of animals are regular diet (RD), high-fat diet (HFD), LI85008F-100 and LI85008F-250, respectively, as described in the Materials and Methods. *, # and $ indicate significance (*P* < 0.05) in RD vs. HFD, HFD vs. LI85008F-100, and HFD vs. LI85008F-250 comparison analysis, respectively, using one-way ANOVA

Figure 3a presents the immunoblot images showing the modulations of essential adipogenic marker proteins in the inguinal fat tissues of the experimental animals. The white adipose tissue (WAT) of LI85008F-250 (G4) rats expressed significant (*P* < 0.05) reductions of PPARγ and its downstream effector proteins C/EBPα, CD36, aP-2α, and perilipin compared to the HFD rats (Fig. 3a). WAT of the LI85008F-100 rats also expressed significantly less (*P* < 0.05) PPARγ, aP-2α, and perilipin, compared with HFD rats (Fig. 3a).

Figure 3b shows the modulation of UCP-1 protein expression in the inguinal WAT of different groups of rats. In comparison with the HFD rats, LI85008F-100 and LI85008F-250 rats expressed 27.80% (*P* = 0.2041) and 49.98% (*P* < 0.05) increases in UCP-1 expression, respectively (Fig. 3b).

### LI85008F modulates serum biochemical indices

**Fig. 4:**
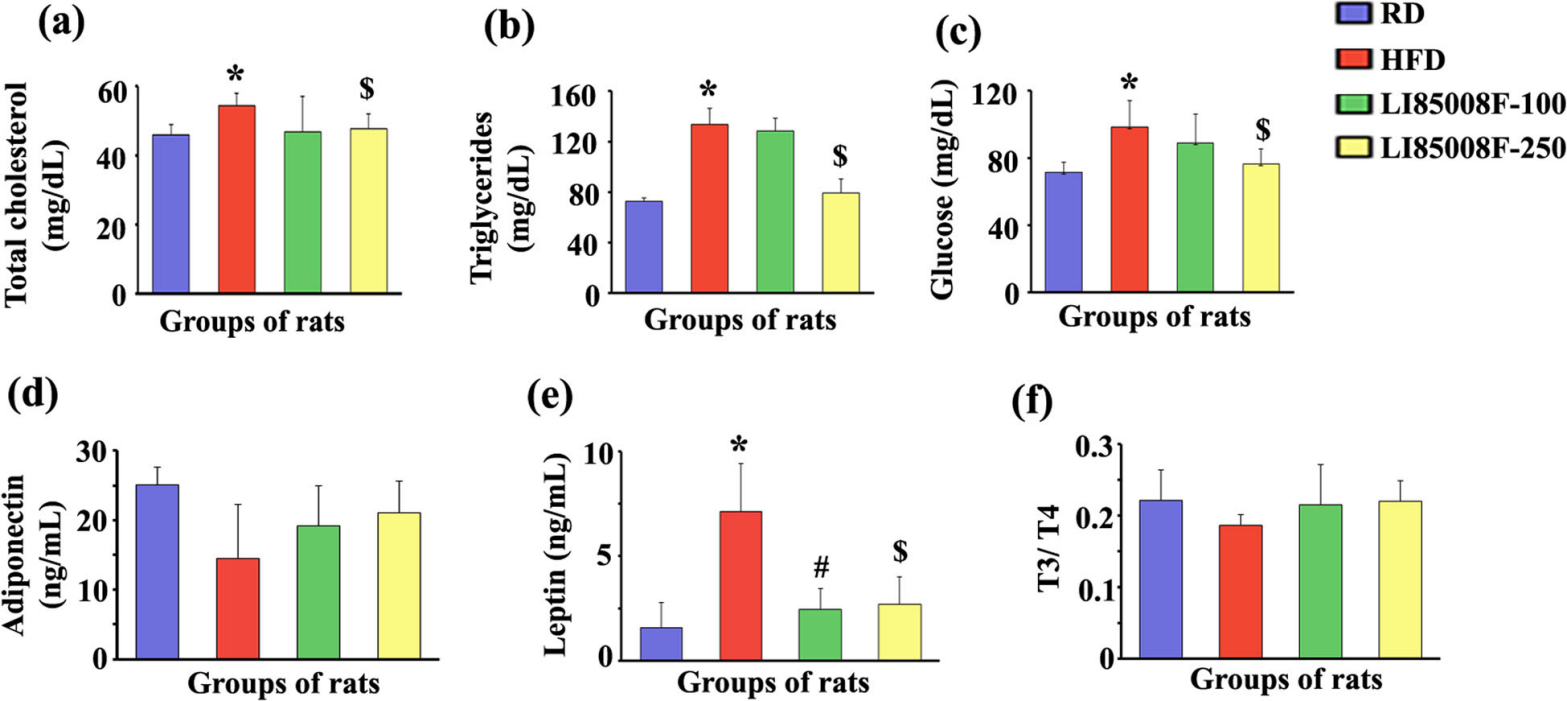
LI85008F improves serum metabolic and endocrine markers in high-fat diet-fed obese rats. Bar diagrams present the mean ± SD of total cholesterol (**a**), triglycerides (**b**), glucose (**c**), adiponectin (**d**), leptin (**e**), and T3/T4 ratio (**f**) in serum samples of the experimental groups of rats; regular diet (RD), high-fat diet (HFD), LI85008F-100 and LI85008F-250, respectively. *n* = 7; *, #, and $ indicate significance (*P* < 0.05) in RD vs. HFD, HFD vs. LI85008F-100, and HFD vs. LI85008F-250 comparison analysis, respectively using one-way ANOVA

The serum TC, TG, and GL levels in HFD rats were significantly higher than those of the RD rats. LI85008F-250 rats showed significant decreases in serum TC (12.34%), TG (40.53%), and GL (22.29%), in comparison with the HFD rats (Fig. 4a-c). LI85008F-100 rats also showed 13.65, 3.64, and 9.55% reductions in TC, TG, and GL levels when compared with the HFD rats, these changes were not significant (Fig. 4a-c).

The herbal blend supplementation did not significantly alter the serum adiponectin concentration, compared with the HFD rats. In the HFD rats, the serum adiponectin level reduced to 57.78%, while the adiponectin level was considered as 100% in RD rats. In LI85008F-100 and LI85008F-250 rats, the improved adiponectin levels were at 76.47 and 83.97%, respectively (Fig. 4d).

Figure 4e shows that the herbal composition supplemented rats significantly reduced serum leptin levels compared with the HFD rats. However, the leptin levels in the LI85008F supplemented rats were not statistically different from the RD rats (Fig. 4e).

Figure 4f illustrates the modulation of the T3/T4 balance in the obese rats. The HFD rats showed a reduced T3/T4 ratio than the RD rats. Both doses of LI85008F supplemented rats showed improved hormonal balance in the obese rats. Compared to RD rats, the changes due to high-fat diet feeding or LI85008F supplementation was not significant (Fig. 4f).

### LI85008F protects from fatty liver development in the HFD-fed obese rats

**Fig. 5:**
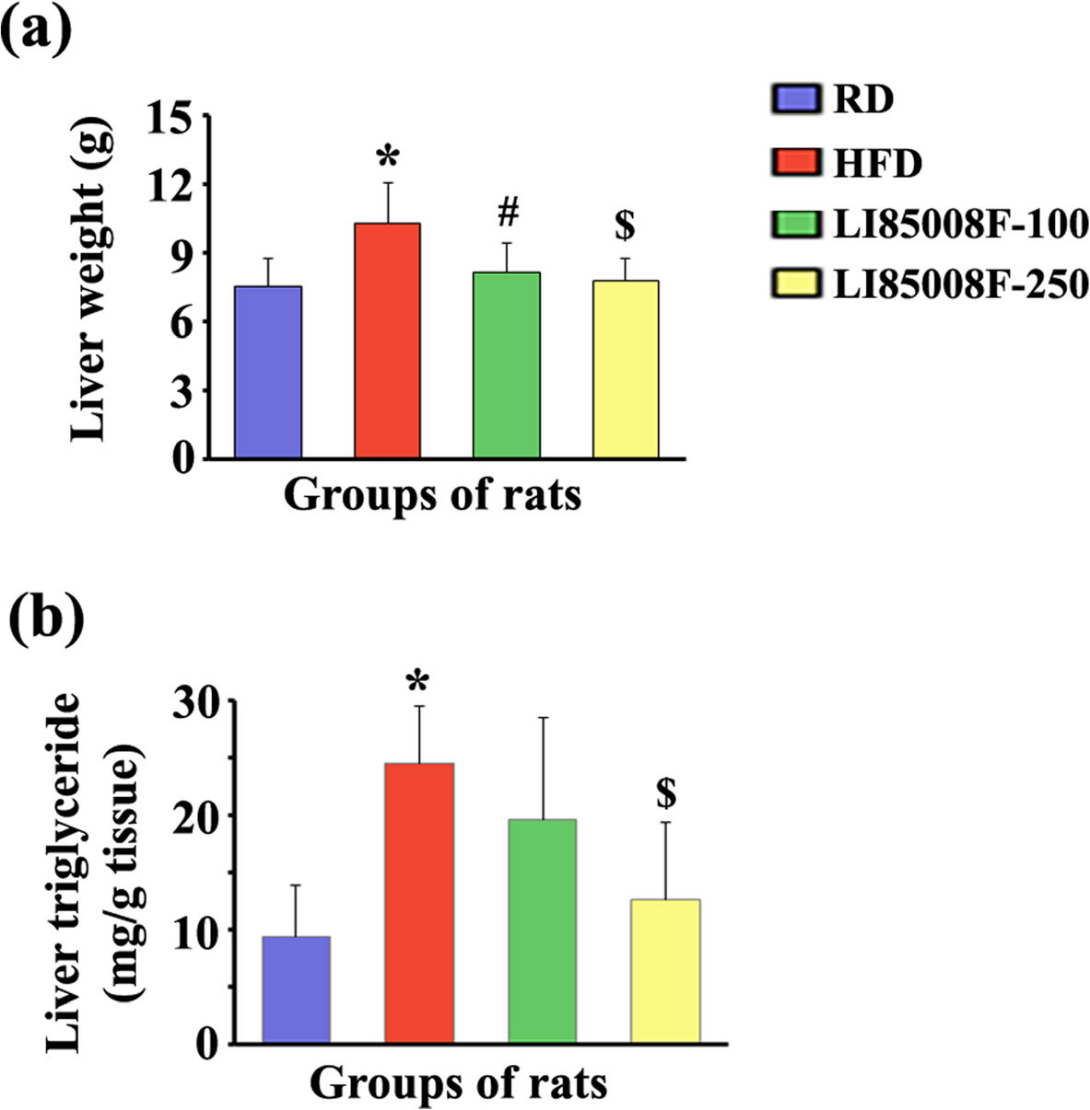
LI85008F supplementation prevents liver enlargement and reduces liver triglyceride concentration in high-fat diet-fed obese rats. Bar graphs present changes in liver weight (**a**) and liver triglyceride (**b**) in the LI85008F supplemented rats. The groups of animals are regular diet (RD), high-fat diet (HFD), LI85008F-100 and LI85008F-250, respectively, as described in the Materials and Methods. n = 7; *, #, and $ indicate significance (*P* < 0.05) in RD vs. HFD, HFD vs. LI85008F-100, and HFD vs. LI85008F-250 comparison analysis, respectively using one-way ANOVA

Figure 5 illustrates that LI85008F supplementation attenuated the increased liver weight and hepatic TG in HFD rats. In the HFD rats, the liver weight increased by 36.96% (*P* < 0.05) in comparison with RD rats. Whereas, LI85008F-100 and LI85008F-250 rats showed 20.77% (*P* < 0.05) and 24.36% (*P* < 0.05) reductions in the liver weight, respectively compared to the HFD rats (Fig. 5a). In parallel, LI85008F-100 and − 250 rats also showed dose-dependent reductions of 19.87% (*P* = 0.3021) and 48.48% (*P* < 0.05) of hepatic TG, respectively, compared to the HFD rats (Fig. 5b).

## Discussion

The present observation provides evidence that LI85008F reduces adiposity in association with increased energy metabolism in high-fat diet-fed obese rats. This study also presents the data on fat cell markers that strengthens the earlier observation of reduced-fat cell formation, which helps explain the weight and fat loss efficacy of LI85008F in overweight and obese subjects [9–11]. Further, this herbal blend influenced energy metabolism to support its weight and fat loss efficacy in HFD-fed obese rats. The amount of energy expenditure at rest or REE contributes more than two-thirds of the daily energy expenditure [17]. Total daily energy expenditure by the body is crucial for maintaining energy homeostasis and healthy body composition [18].

The highlight of the present study is that the combined herbal extract supplementation increased resting energy expenditure in association with reduced body fat mass and prevented BW gain in obese rats. Interestingly, the BW growth pattern in the low dose of LI85008F supplemented rats was marginally close to that of the RD fed rats. This observation suggests that a daily dose of 100 mg LI85008F is adequate to maintain the healthy BW in HFD fed rats. The data also presents an increased expression of an essential thermoregulatory protein, UCP-1, in the inguinal white adipose tissue (WAT) of the herbal supplemented rats. These observations indicate an influence of LI85008F in increasing energy metabolism via browning of WAT, suggesting a basis of decreased adiposity in LI85008F-supplemented obese rats. The up-regulation of UCP-1 is crucial for the thermogenesis process [19]. Recent observations indicate that besides the classical brown adipose tissue (BAT), beige fat cells also influence thermogenesis and reduce body fat mass in rodents and humans [20, 21]. Beige fat cells possess mixed physiological and morphological features of the brown fat cells. These metabolically active cells generate from WAT cells primarily through beta-adrenergic receptor activation in an autocrine, paracrine, or endocrine mechanism when exposed to cold or food ingredients [20, 22, 23]. The browning of WAT requires the active involvement of several metabolic factors that include adiponectin [12, 13, 24] and triiodothyronine (T3) [25, 26].

In the present study, LI85008F supplementation increased the serum adiponectin level, but the effect was not significant. The present observation is not consistent with the earlier clinical study data [10, 11]. One critical observation is the large variability in the adiponectin level in the HFD fed obese group. The effect of LI85008F supplementation could have been significant in larger group sizes. Another interesting observation from the present study is that the improvements in the serum T3 level in the herbal blend supplemented rats. The changes in T3/T4 balance were not statistically significant, but upon LI85008F supplementation, the T3/T4 level was improved to the level of non-obese rats. T3 is the metabolically active form of the thyroid hormone; it increases energy expenditure through lipid and carbohydrate metabolism [27]. The β3AR expression in the inguinal fat tissue of LI85008F rats was unaltered, compared with the obese rats (data not shown). This observation is in agreement with an earlier finding that the involvement of β3AR is not essential for metabolic activation during the fat browning process [28]. In contrast, Lasar et al. reported that PPARγ is required to activate the BAT through β3AR signaling [29]. Collectively, the data suggest that LI85008F increases energy expenditure by modulating the endocrine factors of energy metabolism and does not involve PPARγ dependent β3AR signaling.

In the onset and progression of obesity, new fat cells generated from the fibroblast precursor cells and intracellular lipid accumulation to produce bulky fat cells are essential [30]. The present observations on the modulations of PPARγ and its downstream effector protein expression in the WAT substantiate the earlier in vitro findings [9]. The observations on decreased fat cell size and reduced body fat mass in the herbal blend supplemented rats are noteworthy. These findings provide strong support and partly explain the molecular basis of weight loss efficacy in obese and overweight subjects [10, 11]. Further, LI85008F supplemented rats expressed a substantial reduction of the abdominal fat mass than the HFD-fed obese rats. This observation is in agreement with the earlier clinical observations where LI85008F supplemented overweight and obese subjects demonstrated reduced waist/hip ratio (WHR) vs. placebo [10, 11]. Several clinical studies suggest that increased WHR is a hallmark feature of abdominal obesity and an important indicator of metabolic dysfunction [31, 32].

Abdominal obesity resulting from a sedentary lifestyle in combination with a fatty diet is a potential risk factor for non-alcoholic fatty liver disease (NAFLD), with typical characteristics of elevated levels of triglyceride and cholesterol in circulation associated with an excessive triglyceride accumulation in the liver [33, 34]. It is of note that LI85008F supplementation helped recover the liver mass and liver TG along with improved serum metabolic parameters in the HFD obese rats. HFD fed obese rodents, and obese subjects with fatty liver symptoms exhibit reduced serum adiponectin levels relative to the respective normal healthy controls [35, 36]. In the present study, the parallel observations on the modulations of serum adiponectin and decreases in serum metabolic parameters associated with hepatic triglyceride levels in LI85008F supplemented obese rats are noteworthy. Together, these data suggest a potential role of this herbal blend in preventing fatty liver symptom development in high-fat diet-fed rats.

Dietary ingredients enhance energy metabolism through glucose and lipid metabolism in the body and have shown potential in reducing adiposity and related metabolic dysfunction. A large number of bioactive flavonoids or polyphenols activate the WAT browning process or enhance BAT activity to increase thermogenesis in vitro and in vivo [20, 37]. In both HFD-induced obese mice and cell models, Quercetin remodeled the characteristics of white fat cells to brown-like fat cells via modulating FGF-21, PRDM-16, PGC1α, and UCP-1 [38, 39]. The polyphenol curcumin, from *Curcuma longa* tuber, increased energy expenditure and thermogenesis in mice upon cold exposure through UCP-1 up-regulation in BAT, via dependent, and independent of PPAR activation [40].

### Study strength and limitations

**Fig. 6:**
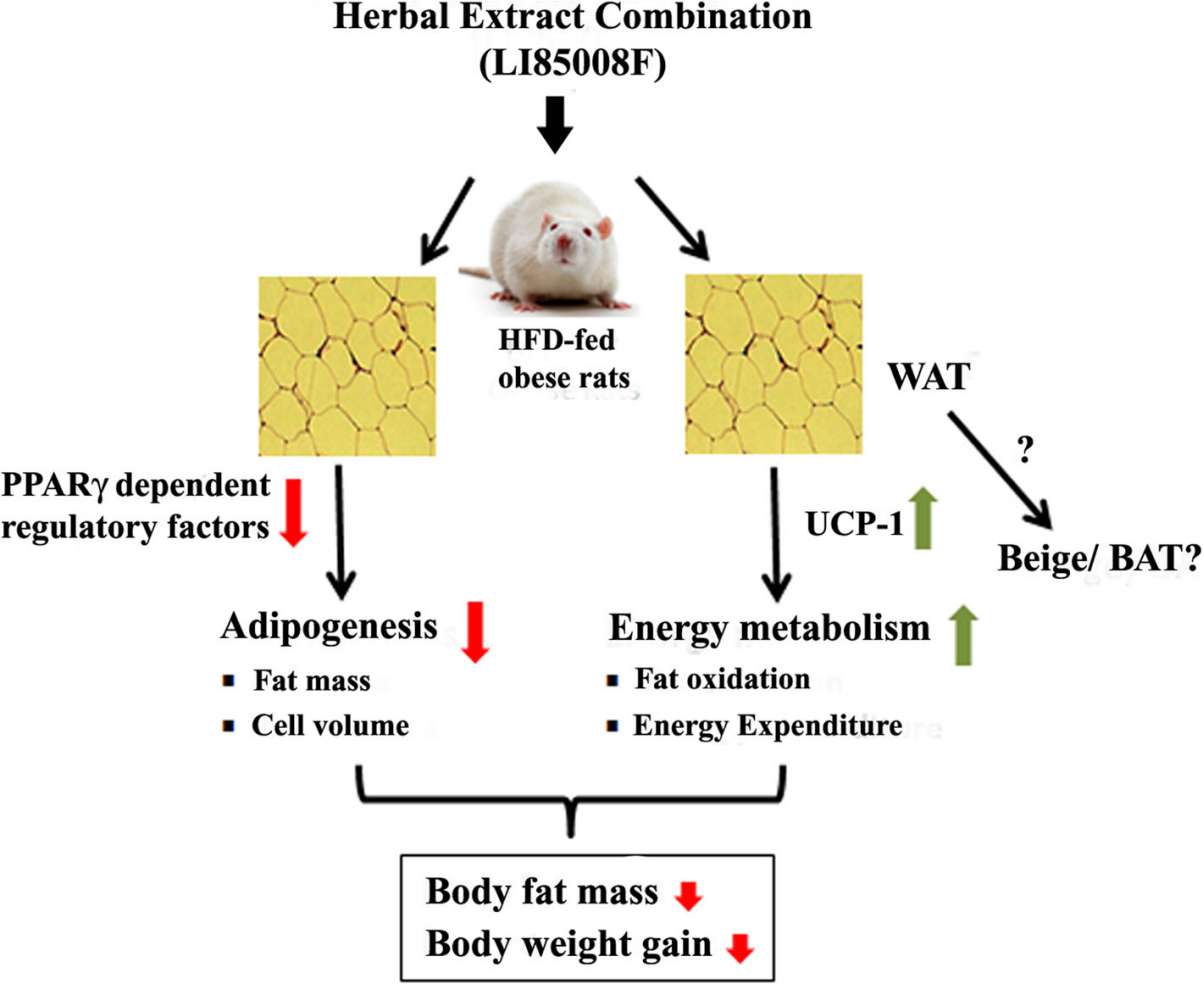
Possible mechanisms of action of LI85008F. The herbal extract combination, LI85008F, down-regulates adipogenesis, and, in parallel, activates energy metabolism in white fat tissue (WAT) of the obese rats. PPARγ-dependent transcription factors include C/EBPα, CCAAT enhancer-binding protein alpha; aP-2α, Activating Protein 2α; CD36, cluster of differentiation 36 and perilipin. PPARγ, Peroxisome proliferator-activated receptor gamma; UCP-1, Uncoupling protein-1. The down headed arrows in red color indicate suppression or reductions; the up arrows in green color indicate up-regulation or increase of metabolic function. The symbol (?) indicates a suggested WAT browning, warrants further exploration

The major finding of the present study is that the combined herbal extracts increased resting energy expenditure in association with reduced body fat mass and prevented BW gain in obese rats. The data also suggest a metabolic link between increased energy expenditure through UCP-1 overexpression in WAT and demonstrates the anti-obesity efficacy of LI85008F in HFD fed obese rats. Figure 6 illustrates the possible mechanisms of action of this herbal blend LI85008F in controlling body weight gain and body fat mass in the HFD-fed obese rats. However, this study has a few limitations. The present data do not explain whether this botanical extract composition influences WAT browning or activates the BAT to increase energy metabolism. Assessment of beige or brown fat cell-specific markers in the fat cells could have answered the query. Another limitation is that the present observation does not provide information on whether LI85008F supplementation confers a benefit of improving insulin sensitivity in obese rats. However, the data on adiponectin and reduced glucose levels suggest a significant role of LI85008F in improving insulin sensitivity in the HFD obese rats. Physical exercise increases energy metabolism through enhanced mitochondrial activity in WAT, alters adipokines secretions, enhances BAT recruitment, and activation [41, 42]. In agreement, evaluating the effect of LI85008F supplementation on energy metabolism in overweight or obese subjects in combination with an exercise schedule is warranted.

## Conclusion

In conclusion, the present observations suggest that LI85008F increases resting energy expenditure via increasing carbohydrate and fat oxidation in HFD fed obese rats. LI85008F increases energy metabolism via UCP-1 up-regulation in WAT through modulation of essential endocrine factors. LI85008F also improves the metabolic markers in serum and liver, indicative of possible protection from HFD-induced fatty liver and clinical symptoms of metabolic dysfunction.

## Data Availability

The data generated in the study are analyzed and presented in this article. The raw data are available on request.

## Abbreviations

ALT: Alanine aminotransferase
AST: Aspartate aminotransferase
BAT: Brown adipose tissue
BMI: Body Mass Index
BW: Body weight
CGMP: Current Good Manufacturing Practice
CMC: Carboxymethylcellulose Sodium
ELISA: Enzyme-linked immunosorbent assay
HFD: High-fat diet
PPARγ: Peroxisome Proliferator-Activated Receptor gamma
RD: Regular diet
RER: Respiratory exchange ratio
REE: Resting energy expenditure
T3: Triiodothyronine
T4: Thyroxin
UCP-1: Uncoupling protein-1
WAT: White adipose tissue
